# Self-care strategies among Arabic-speaking refugees and the Australian Mental Health Stepped Care Model: a Delphi consensus study

**DOI:** 10.1186/s12913-026-14344-1

**Published:** 2026-03-16

**Authors:** Deena Mehjabeen, Ilse Blignault, Nicola Reavley, Shameran Slewa-Younan

**Affiliations:** 1 Translational Health Research Institute, School of Medicine, Western Sydney University, Campbelltown, Australia; 2 Centre for Mental Health, Melbourne School of Population and Global Health, University of Melbourne, Melbourne, Australia

**Keywords:** Access to mental health care, Mental health promotion, Help-seeking, Self-care, Stepped care, Migrant, Refugee, Cultural adaptation, Community engagement

## Abstract

**Background:**

Arabic-speaking refugees and migrants experience elevated levels of anxiety, depression and posttraumatic stress disorder, compared to the host country general population. This study sought to establish consensus on the perceived relevance of different mental health self-care strategies for Arabic-speaking refugees across the four settings of the Australian Mental Health Stepped Care Model: informal community care, primary/generalist care, community-based mental health services, and hospital-based mental health services (formal services).

**Methods:**

The three-round Delphi study engaged a mixed panel of Arabic-speaking community members recruited from South Western Sydney and health professionals (*N* = 42). Data were collected via an online questionnaire, which was administered to the community members in face-to-face groups. Participants were asked to consider 73 self-care activities across three rounds. Activities reaching ≥ 75% endorsement were considered to have achieved consensus, those with 65–74% agreement were re-rated in the next round, and those with < 65% agreement were excluded.

**Results:**

The community panellists were predominantly older women. Consensus was reached on 46 of the 73 activities considered. Forty-five were endorsed for informal community care, 28 for primary/generalist care, 18 for community-based mental health services, and 4 for hospital-based mental health services. Three lifestyle-related activities (eating a balanced and nutritious diet, attending to personal hygiene and connecting with nature) were endorsed across all four settings, indicating their perceived universal relevance. Overall, activities emphasising social engagement, religious and spiritual practices, and culturally familiar community and leisure activities, were strongly favoured in informal contexts. Psychological strategies, such as goal setting, mindfulness and cognitive reframing, were seen as relevant to both informal community care and formal services.

**Conclusions:**

Most culturally and community-grounded mental health self-care strategies for Arabic-speakers with a refugee-like background were found to align with the stepped care framework. These findings can be used as recommendations for practice and program development with focus on integrating these strategies across different settings in collaboration with community organisations and services, and health professionals. Further research is required to examine their relevance for different population subgroups, and their effectiveness and feasibility for integration into clinical care to help address persistent mental health inequities.

**Supplementary Information:**

The online version contains supplementary material available at 10.1186/s12913-026-14344-1.

## Introduction

Globally, refugees and migrants (many of whom have refugee-like backgrounds in that they have experienced pre-migration trauma and displacement) face disproportionately high rates of mental disorders, in particular anxiety, depression and posttraumatic stress disorder (PTSD) [1, 2]. These burdens are exacerbated by adverse social and economic circumstances, including unstable housing, unemployment, social isolation, discrimination and difficulties accessing healthcare [3]. Access to and utilisation of mental health services remain limited [3].

Over the past decade, conflicts and political upheaval in the Middle East and North Africa have resulted in Arabic speakers accounting for a large proportion of the global refugee flow, including over 6.4 million refugees from Syria [4]. Arabic speakers often have negative attitudes towards formal help-seeking, preferring informal help sources such as self-care strategies, including spending time with family and friends and engaging in religious practices [5]. Where services are available, structural and individual barriers related to access and affordability often prevent individuals and families from engaging with care services [5]. For immigrant communities in general, access challenges are compounded by language and cultural barriers, contributing to persistent unmet needs and deepening existing mental health inequities [2, 3].

Self-care, as defined by the World Health Organization (WHO), encompasses the ability of individuals, families and communities to promote health, prevent disease, maintain health and cope with illness and disability, with or without the support of a healthcare provider [6]. Globally, self-care is recognised as a critical strategy for reducing the treatment gap, particularly in low-resource and high-need contexts and for underserved populations such as immigrants [7]. While the specific form of self-care strategies is shaped by social, cultural and religious contexts, it has gained increasing attention as a cost-effective approach to support mental health and wellbeing, both from a systems perspective and a user perspective [6, 8]. By enabling individuals and communities to manage aspects of their mental health outside of formal clinical settings, self-care aligns with international calls to expand scalable, culturally responsive, and sustainable approaches to mental health promotion. Culturally-grounded, self-care strategies can empower immigrant communities, complement formal healthcare services, and reduce the demand on overburdened clinical settings [9].

In the WHO pyramid model for an optimal mix of services for mental health [6, 10], self-care is placed at the bottom. It is the foundation upon which other services, including informal community care, are based. Moving up the pyramid, engagement with formal services and the intensity and cost of interventions increase. In keeping with this model, the WHO has developed a range of online and in-person self-care interventions that incorporate scalable psychological strategies for refugee populations [11]. Interventions such as Problem Management Plus (PM+), EASE and Step-by-Step have been successfully tailored for Arabic-speaking refugees and migrants in transit and resettlement countries [12–14], with evaluations demonstrating their potential impact on the mental health system [12, 14, 15].

Australia is an ‘immigration nation’ established on the lands of Aboriginal and Torres Strait Islander peoples, and a leading destination for migrants and refugees [16]. At the 2021 Census, 29.3% of Australia’s population (around 7.5 million people) were born overseas, and another 22.2% had one or both parents who were born overseas [17]. Arabic-speakers comprised 1.4% of the total population (367,159 people), with Arabic being the second most common language spoken at home, after English and Mandarin [16]. Over half (58.6%) of all Arabic speakers were born overseas; the most common birth countries being Lebanon (20.4%), Iraq (12.0%), Egypt (7.6%) and Syria (5.3%) [17]. Over half (53.9%) identified as Muslim and 21.2% as Catholic Christian [18]. Arabic-speaking Australians predominantly live in Sydney and Melbourne, Australia’s two largest cities, and a substantial proportion have refugee or refugee-like backgrounds [19].

The Australian health system is made up of a diverse network of service providers and health professionals operating across various organisations [20]. It includes both government-funded services at the national, state and territory levels, as well as private sector providers. Together, these entities collaborate to address the physical and mental health needs of the Australian population [21]. Medicare, Australia’s publicly funded universal health insurance scheme, serves as the cornerstone of the public health system, offering subsidised access to general practitioners (GPs), specialists, hospital care and mental health support [20]. Nevertheless, out-of-pocket costs and long wait times can create barriers; issues compounded for migrants and refugees who may also face language, cultural and systemic challenges when seeking care [22].

The Australian Mental Health Stepped Care Model provides a framework for delivering care proportionate to individuals’ needs. It comprises four levels: informal community care, primary/generalist care, community-based mental health services, and hospital-based (tertiary) mental health services [6]. Informal community care refers to health-promoting activities or practices undertaken by individuals, families, and community members, with or without support or advice from health professionals, including use of web-based tools. Primary/generalist care is delivered by GPs and other health professionals; it includes basic healthcare services, preventive care, health assessments and counselling. Community-based mental health services are delivered by mental health professionals or teams who provide specialised care outside hospitals, such as in health clinics, patients’ homes, residential care and assisted living facilities. Hospital-based mental health services provide highly specialised treatment in hospitals or specialised clinics; they are used only when necessary. Outside the health sector, settlement services and non-governmental organisations play an important role by providing culturally appropriate mental health and wellbeing support, facilitating access to care for refugees and assisting with social integration.

Given the need for structured, culturally grounded guidance that aligns with local service systems, the present study sought to establish consensus on the perceived relevance of various self-care strategies used by Arabic-speaking migrants with refugee-like backgrounds (hereafter referred to as ‘refugees’ for brevity) across the different levels and settings encompassed by the Australian Mental Health Stepped Care Model. It employed a rigorous Delphi process, independently recruiting health professionals and Arabic-speaking community members to a combined expert panel, and built on findings from a comprehensive mixed-methods systematic review of the global literature on positive mental health self-care strategies among Arabic-speaking refugees and migrants [9] and a qualitative study that explored the use of and barriers to self-care strategies among 21 Arabic-speaking individuals with refugee backgrounds in South Western Sydney through four online focus group discussions [23]. Both studies identified a wide range of self-care strategies that were grouped into four broad categories: social, psychological, religious/spiritual and other (e.g., expressive arts, exercise, hobbies).

Consensus methods can provide a basis for decision-making and action in circumstances where there is limited evidence [24]. The Delphi process is an iterative method used to assess expert consensus, and especially valuable for complex or under-researched topics [8]. The ability to incorporate diverse stakeholder perspectives makes it well suited for minority mental health research where cultural sensitivity and community engagement are essential, and for developing culturally relevant, practical recommendations that address the unique needs of immigrant populations [24]. Over the years, several Delphi variants and modifications have been developed for different purposes, each with specific benefits and challenges. Common features include an iterative survey (at least two rounds) of experts who bring specialised knowledge (e.g., professional, academic or experiential), a structured communication process, and systematic data analysis [25].

Using the Delphi process, this study sought consensus on ‘What is the perceived relevance of culturally appropriate mental health self-care strategies for Arabic-speaking refugees within the Australian Mental Health Stepped Care Model?’ Strategies were considered across the four settings: informal community care (with or without professional support), primary/generalist care, community-based mental health services, and hospital-based mental health services.

## Methods

### Ethics and consent

Ethics approval was obtained from the Western Sydney University Human Research Ethics Committee (H15581). Participation in the Delphi study was voluntary. All panel members provided informed consent, either electronically or via paper-based forms. Community participants received a $60 grocery voucher.

### Research team

The interdisciplinary research team included representatives from psychology (with specialist expertise in transcultural mental health and mental health promotion, and experience working with the target population) and global public health (including migrant and refugee health).

The study was conducted in collaboration with the Arab Council Australia; a secular, non-profit independent community organisation based in Sydney.

### Delphi procedure

The study followed the four key stages of the Delphi process: formulating the research question; recruiting an expert panel; developing and administering the questionnaire; and analysing responses over three iterative rounds [11]. Data were collected over a 4-month period, from May to August 2024.

### Panel recruitment

We considered as experts both health professionals and Arabic-speaking community members with relevant lived experience. The Delphi panel was drawn from two sources: (1) Arabic-speaking adults (18 years and older) who had migrated to Australia to flee conflict or persecution and had experienced feelings of stress; and (2) qualified health professionals with at least two years full-time or equivalent part-time experience in their discipline and experience working with Arabic-speaking individuals from refugee backgrounds. Although panel sizes for Delphi studies can vary considerably [26], it is generally regarded as suitable to have 15 to 30 participants for a heterogenous panel [27–29]. Our aim was to invite 25 participants for each group to allow for dropouts.

Recruitment materials were distributed via email, word-of-mouth and community sessions. Community participation was assisted by a senior worker from the Arab Council Australia, fluent in Arabic and English, who promoted the study through community networks and at regular group sessions held in a public library in South Western Sydney. The promotional flyer used the term ‘stress’ to foster broader engagement and reduce potential stigma associated with terms like ‘mental illness’ in the target population [30]. Potential health professional participants were identified through their involvement with key government and non-government refugee health organisations and networks, professional colleges and associations, universities and research centres, and personal networks. Identified individuals were sent an email invitation with detailed study information and the Participant Information Sheet (PIS) and encouraged to share it with colleagues. Those who expressed interest in joining the study were emailed the consent form to sign and return.

All study materials (including PIS, consent form and questionnaire) were initially translated into Arabic for the community panellists using Google Translate. The translations were reviewed for accuracy by a bilingual research assistant, and for cultural appropriateness and suitability by the bilingual senior worker from the Arab Council Australia.

### Questionnaire development and administration

The first round of the questionnaire included 67 items which were derived from the comprehensive systematic review examining positive mental health self-care strategies targeting symptoms of PTSD, generalised anxiety, and depression among Arabic-speaking refugees and migrants [9] and a qualitative study involving online focus groups with Arabic-speaking community members of refugee-like backgrounds in South Western Sydney, which explored self-care strategies for managing stress [23]. Including the qualitative findings ensured that the items were not only evidence-based but also locally relevant, enhancing the rigour of the Delphi process. For the purpose of administration, all strategies were re-phrased as activity statements. For each self-care activity, participants were asked to its relevance across the four settings of the stepped care model using a 4-point Likert scale: ‘Very relevant’, ‘Relevant’, ‘Not relevant’ and ‘Don’t know’. At the conclusion of Round 1, participants were given the opportunity to suggest other self-care activities for inclusion in the questionnaire. An additional 13 activities were suggested by the health professionals. Following consideration by the research team, six that were deemed actionable were drafted into new items for inclusion in Round 2.

The questionnaire was administered via Qualtrics, an online survey platform [31], and piloted to test comprehension and ease of completion. It was divided into four sections: demographic data collection; explanations of self-care and the stepped care model; the survey instructions; and the survey items, each accompanied by pictorial image. The items were not grouped in any way. In the first round, participants were also invited to provide their comments on any self-care activity that they thought should be in the survey but was not currently included. Other than the demographic section, the content was the same for both groups of panellists. An English version of the Round 1 questionnaire for community members is provided in the supplementary materials.

In each round, the survey was administered to community members and health professionals, who were asked to complete it within the same 2-week window. Community panellists were administered the Arabic version of the questionnaire in a group session held in a familiar community setting (public library), with refreshments provided and childcare support if needed. They were supported by the bilingual group facilitator or peers and were given the option of paper and digital formats (via QR code). Laptops and tablets were made available during the sessions for those without internet-enabled mobile phones. Some participants chose to take the paper questionnaires home to complete them at their own pace, with between 3 and 21 individuals taking this option across the rounds. Health professional panellists completed the English version of the questionnaire online via Qualtrics. Email and text message reminders to complete and return the questionnaires were sent in Arabic for community panellists, and in English for health professional panellists.

**Fig. 1:**
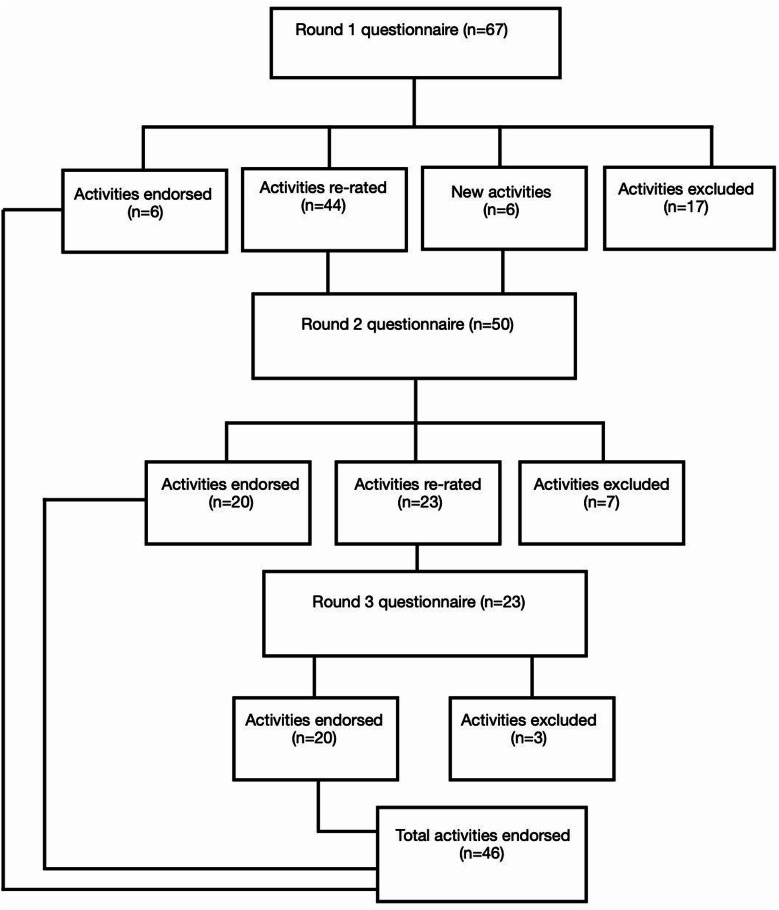
Number and outcomes of activities in each Delphi round

For the second and third rounds, items that had reached consensus (either endorsed or excluded) were removed, and panellists were presented only with the remaining items for re-rating, allowing them to further reconsider their judgements. After Round 1, six new items were added based on suggestions from health professionals (see Fig. 1); these were selected because they were not already represented in the survey and could be phrased in an actionable manner. Thus, the questionnaire was progressively shortened across the three rounds (67 items, requiring approximately 60 min to complete; 50 items; and 23 items), reducing the time needed to finish. No changes were made to the wording of existing items, and instructions at the beginning of round 2 and 3 surveys explained the purpose was to re-rate ambiguous items and, in the case of round 2, to consider newly suggested items.

### Analysis

The quantitative responses were analysed using descriptive statistics. Consensus was defined in terms of percentage agreement. In each round, the same criteria were applied for endorsing, excluding and re-rating activities. Activities that did not meet endorsement criteria in any of the four settings were excluded. Activities that required re-rating from Round 1 went into Round 2 together with the new items, and those that required re-rating from Round 2 went into Round 3. For the combined panel, thresholds were defined as follows: activities with ≥ 75% agreement were endorsed, those with 65–74% agreement were re-rated in the next round, and those with less than 65% agreement were excluded [32]. Due to unequal numbers in each panellist group, specifically the health professionals group having fewer than 25 members, responses were weighted to ensure balanced representation between community members and health professionals. Weighting involved calculating an overall endorsement percentage based on the relative sizes of the participant groups. The proportion of the total sample for each group was determined, and the endorsement percentage within each group was multiplied by its respective proportion. These weighted values were summed to produce the final endorsement percentage, which was then compared with the predefined consensus thresholds.

### Rigour and community feedback

The process of drafting and phrasing of activity statements for the questionnaire was undertaken by the research team. Quantitative analysis of the successive survey rounds was undertaken by at least two team members. Findings from the earlier qualitative study supported interpretation of community panellists’ responses, enhancing the rigour of the Delphi process. We followed the recommendations for interdisciplinary standardised reporting of Delphi studies (DELPHISTAR) in preparing this article [25].

Following completion of the research, a 2-page infographic based on the Delphi study findings and including a list of community resources, was prepared and distributed to all study participants.

## Results

### Demographic characteristics

The 42 expert panellists included both Arabic-speaking community members and health professionals. Table 1 presents the demographic characteristics of the two groups.

The 28 community panellists (25 women and 3 men) all resided in South Western Sydney. Most came from Iraq and Lebanon and had lived in Australia for over five years. They included Christians and Muslims. Most were aged 55 years and older and were not in the workforce. Occupations included homemakers, carers, retirees and support workers. Retention was 100% across the first two Delphi rounds, with one dropout in Round 3 due to the participant travelling overseas.

The 15 health professionals (12 women and 3 men) were mostly based in New South Wales (NSW), for which Sydney is the capital city. Their professional backgrounds included psychology, medicine, public health and exercise physiology, and all but one had over five years of experience. Seven spoke only English, seven spoke a language other than English (including Arabic), and one preferred not to answer the language question. Two described themselves as non-religious and four did not disclose their religion. There was full retention over the three Delphi rounds.

### Endorsed activities

In the first Delphi round, 81.6% of the 42 expert panellists agreed on the endorsed activities. In the second round 82.4% agreed, and in the third round 84.9% agreed, demonstrating a small but steady rise in consensus.

Table 2 presents the endorsed activities across the four settings categorised by broad strategy type. Of the 73 self-care activities reviewed (including 67 presented initially and 6 added later), 46 were endorsed by the panel: 45 (61%) for informal care, 28 (38%) for primary/generalist care, 18 (25%) for community-based mental health services, and 4 (6%) for hospital-based mental health services. Thirteen of the endorsed activities were categorised as social strategies, 13 as psychological strategies, 4 as religious strategies and 16 as ‘other’ strategies, consistent with the framework developed and used in the previous studies [9]. The large ‘other’ category was further broken down into four sub-categories: healthy lifestyle, hobbies, acts of service and responsibility, and miscellaneous.

The endorsed social strategies included activities such as spending time with people of the same culture or religion, connecting with neighbours and community members, spending quality time with your partner, playing with children, and seeking help from trusted sources. Psychological strategies included setting and working towards personal and professional goals, practising mindfulness, deep breathing or muscle relaxation, reframing negative thoughts, and being thankful for positive life events. These activities were generally seen as relevant to informal community care and primary/generalist care, with some endorsed for community mental health services as well. The four religious strategies included praying, attending religious services, reading or listening to religious texts, and observing religious rituals.

Among the other strategies, healthy lifestyle behaviours, such as eating a balanced diet, maintaining hygiene connecting with nature, physical activity and adequate sleep, were endorsed across most settings. Hobbies and acts of service and responsibility, such as volunteering and housework, were mainly endorsed for informal community care, although listening to music and gardening were also considered relevant to community-based mental health services.

### Excluded activities

Of the 27 excluded activities, 15 items were excluded by both groups of panellists, 9 by community members and 3 by health professionals (see Table 3). Both groups excluded digital and leisure activities such as browsing the internet and social media, playing electronic games, and watching movies, funny videos or children’s cartoons, as well as eating sweets, having a cold drink to calm oneself and drinking herbal tea. They also excluded certain hobbies, specifically reading or writing poetry, doing different crafts and changing house décor, as well as learning to drive, going for drives and taking train trips, and spending time with pets. Shopping was also excluded.

Other activities, excluded by one group of panellists while receiving endorsement from the other group, highlight their differing perspectives. For example, the exclusion of activities such as keeping a diary to reflect on daily situations in life, finding work and pursuing vocational training, as well as engaging in performing and visual arts, singing in a choir and travelling offer valuable insight into the priorities and preferences of the community panellists, most of whom were middle-aged and elderly women. Practising meditation and seeking help from religious leaders were also excluded. The health professionals excluded going to the cinema, watching television and designing clothes.

### Qualitative responses

Health professionals commented on the challenges associated with practising self-care. For instance, they mentioned that help-seeking within the community might be perceived as risky or even harmful due to concerns about gossip, breaches of confidentiality, and community politics. Additionally, communities may lack the resources or knowledge necessary to adequately support their members. Services themselves may contribute to mental health problems through institutional racism, limited access, and poor communication with service providers. Financial issues, such as the sudden cessation of social security payments, also contribute to psychological distress. It was made clear that like other elements of treatment, including self-care strategies, should be person-centred, recognising that what feels beneficial can vary greatly between individuals. The relationship between religion and gender were cited as examples. Religious strategies may be relevant for some people but not for others. One respondent, who chose ‘Don’t know’ option for such items, explained that while attending religious services may be ‘Very relevant’ for some men, it may not be so for women, depending on religious and cultural norms. Another comment related to excessive use of social media and television, particularly content related to distressing situations in the country of origin, was seen as potentially harmful and something to be actively avoided.

## Discussion

This study aimed to find consensus among community members and clinicians on the relevance of culturally grounded mental health self-care strategies for Arabic-speaking refugees across the four settings encompassed by the Australian Mental Health Stepped Care Model. The activities considered by the combined panel were derived from a comprehensive systematic review that identified self-care strategies practised by Arabic-speaking refugees and migrants globally, together with a qualitative study that generated Australian context-specific perspectives. Similar to the qualitative study participants, the community panellists resided in South Western Sydney, a region with a high refugee intake [33].

Almost two-thirds of the activities presented to them were endorsed by the combined expert panel. All but one were endorsed for informal community care. The single exception was problem solving by writing down the problem and thinking about the best solution (a psychological strategy), which was endorsed for primary/generalist care, suggesting that professional support was seen as helpful in this instance. Over one-third of the activities reviewed were endorsed for primary/generalist care. A quarter were endorsed for community-based mental health services, and four for hospital-based mental health services. These findings indicate a broad acceptance of self-care strategies, not only for informal community care but also in formal healthcare settings—particularly primary/generalist care and community-based mental health services. While many of these may have a social component, they can also be individual or solitary activities.

Following our previous work, we initially grouped the endorsed activities into four broad categories (social, psychological, religious and other), while recognising a degree of overlap (e.g., attending religious services has both a religious and social component, and going to stress management workshops has both a psychological and social component). Social strategies were strongly endorsed. Such strategies align with the values of social connectedness and mutual care found among Middle Eastern cultures [34]. Seeking support from family, friends and community is deeply rooted in collectivist culture and identity [35, 36]. Psychological strategies, especially those that align with cultural and religious practices and support resilience in the face of resettlement challenges were also strongly endorsed. Religious practices such as prayer and ritual observance and attending religious services play a central role in the mental wellbeing of Arabic immigrants, both Christian and Muslim [9], although their relevance may vary by gender (e.g., mosque prayers perceived as more male-oriented) [37]. In addition to connection and a sense of community and identity, faith can provide emotional comfort and stability during times of displacement and hardship [38–41].

Panellists also agreed on inclusion of a large number of ‘other’ strategies that did not fall neatly into one of the first three categories. The endorsement of physical activities and healthy lifestyle habits (sleep, diet, personal hygiene) underscores the connection between physical health and mental health [42]. The mental health benefits of connecting to nature and taking break from digital world (mobile phone and social media) are increasingly being recognised [43–45] and were also endorsed by the panellists. The relevance of these activities across multiple settings highlights the holistic nature of self-care, which spans physical, emotional, social and spiritual wellbeing. Engaging in hobbies, activities undertaken for pleasure in one’s own time and without professional or financial gain, can also contribute to mental health, helping reduce stress and improve mood [46]. Panellists endorsed four hobbies (gardening, cooking and baking, listening to music, reading); however preferences are likely to be gender and age-related, as well as context dependent. Acts of service and volunteering, support individual, family and community wellbeing, by providing opportunities to connect with others and creating a stronger sense of purpose [47, 48]. Learning English is important for successful acculturation, while study may improve employment prospects and support personal growth [49]. Pampering oneself can boost self-esteem and reinforce identity maintenance, particularly among migrant women [50].

Several of the activities excluded by community members only, such as finding work, pursuing vocational training, engaging in performing or visual arts, and travelling, likely reflect the everyday lives and practical considerations of the study participants who were predominantly older women. Such activities will have more relevance for younger members of the Arabic-speaking community. Although practising mindfulness and muscle relaxation were endorsed, practising meditation was excluded; this may reflect the community panellists’ understanding of this term. Seeking help from religious leaders was also excluded. This may reflect recognition that religious figures are not always trained to address mental health problems, or concerns about confidentiality and mental illness stigma [30, 51–53]. The previous qualitative study highlighted the importance of religion and spirituality for emotional support [23]. The systematic review found that although some studies reported people seeking support from religious leaders, others noted a degree of distrust towards them [38]. Similarly, other studies among Arabic-speaking refugees have shown that while religion remains central to coping, individuals often prefer self-directed or family-based approaches over formal religious counselling [54, 55]. Health professionals, but not community members, excluded going to the cinema, watching television and designing clothes. Watching television especially may be seen as passive and without direct mental health benefit. Notably, internet and social media were excluded by both groups. As well as being passive, there is potential for disturbing or harmful content [56]. Indeed, research has indicated that some activities while initially seen as passive or benign in small doses (e.g. watching television or social media use) can become problematic if engaged in frequently [57, 58].

As part of a person-centred approach, self-care is positioned largely as an individual or micro-level practice [8, 59], with an emphasis on individual autonomy and individual capabilities and capacities which are considered Western values. Social and community networks are viewed as having a role in encouraging and supporting self-care, rather than as an essential element. However, given the collectivist values of Arabic-speaking populations and the current findings, a broader approach—one that also incorporates family and community, and faith as appropriate—may be more culturally acceptable and effective. In this, the Delphi study findings align with a recent systematic review of psychological group interventions for adult refugees in resettlement countries [60], which recommends that in-language, culturally adapted group and community interventions should be considered for integration into stepped care models at the lower tiers. Studies in Sydney, Australia, have demonstrated the effectiveness of in-person and online group-based interventions that teach mindfulness skills to Arabic-speakers [61, 62]. Addressing the social determinants of health, such as employment, education and housing, is also integral to supporting mental health self-care and creating the conditions necessary for refugees to thrive in their new home [3].

### Dissemination and implications

The study findings were converted into a simple infographic and disseminated to all participants, with Arabic translations for community members, and shared with the Arab Council Australia. More broadly, the findings were disseminated to health professionals, community organisations and resettlement services through national and local conferences. These activities formed the core of the research translation strategy, ensuring that the results were communicated in culturally appropriate ways and positioned for use in practice and program development.

Arabic-language self-care resources can enhance accessibility and relevance for Arabic-speaking people with refugee-like backgrounds, while addressing stigma and negative perceptions of mental health in these communities [63]. Several resources are available online through the NSW Transcultural Mental Health Centre and the NSW Multicultural Health Communication Service.

Informal settings provide a context for self-care for everybody, particularly for people who may not access formal services and that could serve as first-line, low-intensity approaches for managing stress and supporting wellbeing. Social and culturally meaningful activities, such as spending time with family, connecting with community members and attending cultural events, support cultural continuity, strengthen social networks and enable early, community-led responses to distress. Primary/generalist care, the first of the formal services in the stepped care model, provides an opportunity for problem solving, and bridges informal support and specialist care. In community mental health services, activities such as engaging with cultural or religious communities, listening to music, reframing negative thoughts and practising gratitude can be encouraged. In hospital settings where people are most unwell, personal hygiene, a balanced diet, connecting with nature and practising mindfulness are still relevant.

### Future research

Globally, there is a growing body of evidence that incorporating self-care strategies alongside professional (i.e., clinician-delivered) psychological and social interventions can support mental health and wellbeing [9]. Future research should explore ways in which the self-care strategies identified in this Delphi study can best be integrated into the Australian Mental Health Stepped Care Model. This includes investigating their implementation in different healthcare settings and geographic contexts, and assessing their potential contribution to improved mental health and wellbeing through ongoing evaluation. Further studies are needed with different population subgroups. Arabic-speaking Australians are diverse in terms of their countries of origin and their migration journeys. Our community participants were predominantly older women; thus the findings may not be readily transferable to the broader Arabic-speaking population with refugee-like backgrounds. Future studies should aim to recruit younger community members, including adolescents and young adults, and men, to support age and gender appropriate mental health self-care. These considerations should be taken into account when designing interventions or applying the results in other contexts.

### Strengths and limitations

This study has several strengths that enhance its value in identifying mental health self-care strategies for Arabic-speaking refugees and migrants that could be promoted in different settings. The anonymity and highly-structured approach that define the Delphi method reduce common sources of response bias such as dominance and group conformity [64, 65]. Recruitment of a combined panel ensured that the endorsed self-care activities were both culturally grounded and clinically informed. There was high retention across the rounds, with only one participant dropping out. Support from a high-profile and respected Arab community organisation was key to recruiting and engaging community panellists. Translation of all research materials into Arabic overcame the language barrier for community members with low English proficiency. The face-to-face group sessions were assisted by a bilingual facilitator and offered a range of options for completing the survey, enabling participants to ask questions and addressing issues faced by those with limited digital access or literacy. Basing the initial questionnaire on self-care activities identified through a global systematic review and a qualitative study with Arabic speakers in Sydney ensured the study built on the broad evidence base and was relevant to the Australian context. Additional activities proposed by health professional participants were also considered.

The study also has limitations. The combined panel lacked geographic heterogeneity with most health professionals from NSW and community members from South Western Sydney, which may limit transferability to other Australian jurisdictions and regions. Despite strenuous efforts by the research team, fewer health professionals were recruited than expected. The community members were mostly middle-aged and elderly women, as were the participants in the earlier qualitative study. Some of the endorsed self-care activities may be less suitable or meaningful for younger people or men, or those in the workforce. The lengthy online questionnaire focused on quantitative responses (4-point Likert scale) and did not explore the reasons behind them. Although both groups were invited to provide comments or suggest new activities, only health professionals completed this section. Offering a verbal feedback option might have encouraged more input from community members. We did not employ an accredited translator/interpreter but relied on a bilingual research assistant and community worker for language support, including document translation and group facilitation.

## Conclusion

This study demonstrated the value of inclusive and iterative consensus-building through a Delphi process involving both community members and health professionals. Collaboration between healthcare providers, community leaders, and cultural organisations remains essential to sustain culturally relevant care. The majority of the endorsed self-care strategies were in the informal care setting, but many were also considered to have a place in formal healthcare settings. The results provide valuable insights into the culturally specific self-care practices that are most relevant to this population. Strategies such as connecting with family and friends, engaging in cultural activities, mindfulness practices, physical activity and observing religious activities were consistently endorsed, reflecting the central role of community, culture and faith in the lives of Arabic-speaking individuals from immigrant backgrounds. These findings underline the need for mental health support frameworks that integrate these cultural dimensions into care models, ensuring that mental health interventions are relevant and acceptable.

**Table 1:** Characteristics of panellists

Community members (*n* = 28)		Health professionals (*n* = 15)	
Characteristics	*n* (%)	Characteristics	*n* (%)
Gender		Gender	
Female	25 (89)	Female	12 (80)
Male	3 (11)	Male	3 (20)
Age group (years)		Age group (years)	
25–34	2 (7)	25–34	5 (33)
35–44	3 (11)	35–44	1 (7)
45–54	1 (4)	45–54	2 (13)
55–64	8 (29)	55–64	6 (40)
65+	14 (50)	Undisclosed	1 (7)
Country of birth		Professional background	
Iraq	16 (57)	Psychology	7 (47)
Lebanon	9 (32)	Medicine	5 (33)
Syria	2 (7)	General practice	1 (7)
Undisclosed	1 (4)	Public health and education	1 (7)
		Exercise physiology	1 (7)
Marital status		Professional experience	
Married	19 (68)	2–5 years	1 (7)
Widowed/Divorced	9 (32)	>5 years	14 (93)
Education		Work sector	
School	23 (82)	Government	6 (40)
Foundation course	3 (11)	Other	9 (60)
University	2 (7)		
Occupation		Languages spoken	
Not in the workforce	20 (71)	English only	7 (47)
Paid employment	5 (18)	Arabic and other languages	7 (47)
Undisclosed	3 (11)	Undisclosed	1 (7)
Religion		Religion	
Christian	16 (57)	Christian	6 (40)
Muslim	11 (40)	Muslim	3 (20)
Undisclosed	1 (4)	Non-religious	2 (13)
		Undisclosed	4 (27)
Length of residence Australia			
≤ 6 months	2 (7)		
> 6 months − 1 year	3 (11)		
> 1–5 years	1 (4)		
> 5 years	22 (79)		

**Table 2:** Self-care activities endorsed by panellists

Self-care activity	Setting			
	Informal community care	Primary/generalist care	Community mental health services	Hospital mental health services
**Social strategy**				
Having meaningful conversations with family and friends	R1			
Spending time with people of the same culture or religion	R1	R2	R2	
Connecting with neighbours and community members	R1	R2	R2	
Spending quality time with your partner	R2	R3		
Playing with children	R1			
Discussing problems with friends, family and trusted others	R1	R2		
Making friends	R1	R2	R2	
Asking for help from family, friends and trusted others	R1	R2	R2	
Attending cultural or ethnic festivals and events	R2			
Visiting family and friends	R1	R2		
Going to cafes and restaurants with family or friends	R2			
Going to social gatherings and parties	R2	R3		
Joking and laughing with friends	R1	R2	R3	
**Psychological strategy**				
Setting personal or professional goals and working towards them	R2	R2	R1	
Practising deep breathing	R2			
Practising mindfulness	R1		R3	R3
Taking a break from stressful situations and negative people	R2	R3	R3	
Going to stress management workshops and short courses	R3			
Problem-solving by writing down the problem and thinking about the best solution		R2		
Reframing thoughts into realistic and positive ideas and beliefs	R1	R1	R2	
Spending ‘me’ time	R1	R2		
Learning to accept situations in life that cannot be changed	R2	R2		
Practising muscle relaxation exercises	R1	R2		
Being thankful for positive life events	R1	R2	R2	
Engaging in positive self-talk (e.g., writing positive affirmation statements)	R3			
Accepting diverse perspectives and others’ views	R2	R2		
**Religious strategy**				
Praying	R1			
Attending religious services	R1		R2	
Reading or listening to verses from the holy books or other religious writings	R2		R3	
Observing religious rituals	R2			
**Other strategy**				
***Healthy lifestyle***				
Physical activity (e.g., walking, jogging, yoga, playing sports, cycling, swimming)	R1	R1	R1	
Eating a balanced and nutritious diet	R2	R2	R2	R3
Looking after personal hygiene (e.g. showering, getting dressed in clean clothes)	R2	R2	R3	R3
Sleeping	R1	R2		
Connecting with nature (e.g., parks, beaches, birdwatching, picnics, zoo visits)	R1	R2	R2	R3
Taking breaks from the phone and social media	R2	R3		
***Hobbies***				
Listening to music	R2		R3	
Reading	R2			
Gardening	R1		R3	
Cooking and baking	R1			
***Acts of service and responsibility***				
Volunteering and community service	R1	R2		
Performing acts of service and charity	R1	R3		
Doing housework	R1	R2		
***Miscellaneous***				
Studying and pursuing education	R3	R3		
Learning English	R1			
Pampering oneself (e.g., visiting the hairdresser, nail salon, facials)	R2			

**Table 3:** Self-care activities excluded by panellists

Self-care activity	Excluded by		
	Both groups of panellists	Community members	Health professionals
Changing house décor	X		
Browsing the internet and social media	X		
Playing electronic games (e.g., mobile phone)	X		
Taking train trips	X		
Learning to drive	X		
Going for drives	X		
Reading or writing poetry	X		
Watching movies	X		
Watching comedy shows, funny videos or children’s cartoons	X		
Spending time with pets	X		
Eating sweets	X		
Having a cold drink to calm oneself	X		
Drinking herbal tea	X		
Doing crafts (e.g., making cards, sewing, knitting)	X		
Shopping	X		
Keeping a diary to reflect on daily situations in life		X	
Finding work		X	
Practising meditation		X	
Pursuing performing arts (e.g., acting, storytelling, singing, playing music, dancing)		X	
Pursuing visual arts (e.g., drawing, painting, photography, filmmaking, graphic design)		X	
Singing in a choir		X	
Travelling		X	
Seeking help from religious leaders		X	
Pursuing vocational training (e.g., TAFE courses)		X	
Going to the cinema			X
Designing clothes			X
Watching television			X

## Supplementary Information

Below is the link to the electronic supplementary material.

Supplementary Material 1

## Data Availability

The authors confirm that the data supporting the findings of this study are available within the article and its supplementary information files.

## Abbreviations

GP: General Practitioner
PIS: Participant Information Sheet
PTSD: Posttraumatic Stress Disorder
NSW: New South Wales
WHO: World Health Organization
