# Supplementary material for: Self-care strategies among Arabic-speaking refugees and the Australian Mental Health Stepped Care Model: a Delphi consensus study
Source: BMC Health Serv Res. 2026 Mar 16;26:469. doi: 10.1186/s12913-026-14344-1 (PMC13049987; doi:10.1186/s12913-026-14344-1)
Supplement: Supplementary file 1 — Supplementary Material 1 [file 12913_2026_14344_MOESM1_ESM.pdf]

Delphi_R1_Community Members

Project Title: Recommendations for mental health self-care strategies among Arabic-speaking community members from refugee-like backgrounds living in Australia: A Delphi study.

First Round Delphi Survey

Participant Information Sheet

Project Summary:
This study aims to develop a set of mental health self-care recommendations for Arabic-speaking community from refugee-like backgrounds living in Australia. The purpose is to gather opinions on mental health self-care strategies from two groups of people: health professionals who have experience working with the target group (practitioners and researchers and academics) and Arabic-speaking community members and leaders. You have been invited to participate in the panel comprising Arabic-speaking community members and leaders.

Participants are required to meet the eligibility criteria:

- Are aged 18 years and above
- Speak Arabic
- Are overseas-born and migrated to Australia due to fleeing conflict or persecution in your home country
- Experienced self-reported feelings of stress

What will I be asked to do?
You will be required to complete three rounds of an online survey. Each round will be opened for two weeks.
In each survey round, you will be asked to give your opinion regarding the relevance and suitability of the listed self-care activities for managing stress and improving mental health among Arabic-speaking populations from refugee-like backgrounds, across the four settings in the Australian Mental Health Stepped Care Model. The response options are: 'Very relevant', 'Relevant', 'Not relevant' and 'Do not know'. You may choose multiple settings for a single self-care activity.

Open-ended comment boxes will be available to allow you to provide specific feedback regarding any new self-care activity that is not included in the survey.

How much of my time will I need to give?
Each survey round will take approximately 60 minutes to complete.

Can I withdraw from the study?
Your participation is entirely voluntary and you are not obliged to be involved. If you do participate, you can withdraw at any time without giving reason and without any impact on your relationships with the researchers or partner organisations. However, once the survey round has closed, individual data will no longer be able to be withdrawn from the survey. All survey data will be de-identified.

You can download and read the Participant Information Sheet here.

Do you meet the eligibility criteria?

- Yes
- No

Consent

I hereby consent to participate in the above named research project.

I acknowledge that:

- I have read the participant information sheet and have been given the opportunity to discuss the information and my involvement in the project with the researcher/s.
- The procedures required for the project and the time involved have been explained to me and any questions I have about the project have been answered to my satisfaction.
- I understand that my involvement is confidential and that the information gained during the study may be published and stored for other research use but no information about me will be used in any way that reveals my identity.
- I understand that my participation in this study will have no effect on my relationship with the researcher/s and any organisations involved, now or in the future. I understand that participation is entirely voluntary and I am not obliged to be involved.
- If I do participate, I can withdraw at any time without giving reason. I understand that I am able to withdraw my data and information within the time (two weeks) that each survey is opened and that once the survey closes I will be unable to withdraw my data and information given within that survey round from this project.

Delphi_R1_Community Members

I consent to participate in this project
- Yes
- No

I consent to providing my data to be used in other related projects for an extended period of time
- Yes
- No

Demographic Information

We are collecting your name and email address for sending the subsequent rounds of survey and a summary of results from the previous round. This information will only be accessible to the research team. Once this study is completed, the file containing participants' names and email addresses will be deleted permanently.

First name

Phone number

Email address

What is your age?

- 18–24
- 25–34
- 35–44
- 45–54
- 55–64
- 65 or older
- Prefer not to say

What is your gender?

- Woman
- Man
- Prefer not to say

Which of the following best describes your religious beliefs?

- Muslim
- Christian
- Mandaean
- Other (please specify)
- Prefer not to say

What is your country of birth?

- Country of birth

- Prefer not to say

Delphi_R1_Community Members

How many years have you lived in Australia?

- 6 months or less
- Over 6 months, up to 1 year
- Over 1 year, up to 3 years
- Over 3 years, up to 5 years
- Over 5 years
- Prefer not to say

What is your marital status?

- Never married
- Married
- In a relationship
- Divorced/Separated
- Widowed
- Prefer not to say

What is the highest level of education you have attained?

- Primary
- Secondary
- Tertiary (College/University)
- Did not go to school
- Other (please specify)

- Prefer not to say

What is your occupation and employment status (e.g., full-time, part-time or casual)?

- Occupation and employment status

- Prefer not to say

Introduction

Self-care, as defined by the World Health Organization (WHO) is 'the ability of individuals, families and communities to promote health, prevent disease, maintain health and cope with illness and disability with or without the support of a health worker'.

This survey describes self-care activities and practices that were identified by comprehensively examining the international research evidence and by undertaking focus group discussions with a purposive sample of Arabic-speaking individuals from refugee-like backgrounds residing in South Western Sydney.

We would like to know your opinion regarding the relevance and suitability of promoting each self-care activity within the four settings in the Australian Mental Health Stepped Care Model: (1) informal community care, (2) primary/generalist care, (3) community-based mental health services and (4) hospital-based mental health services.

This information will then be used to achieve the research aim, that is, to develop a set of recommendations for self-care activities and practices to manage stress and improve mental health for Arabic-speaking people from refugee-like backgrounds in Australia.

Delphi_R1_Community Members

Understanding the importance of self-care within the stepped care model is fundamental to completing the survey. As depicted in the figure below, self-care is applicable in each of the four settings within the model.

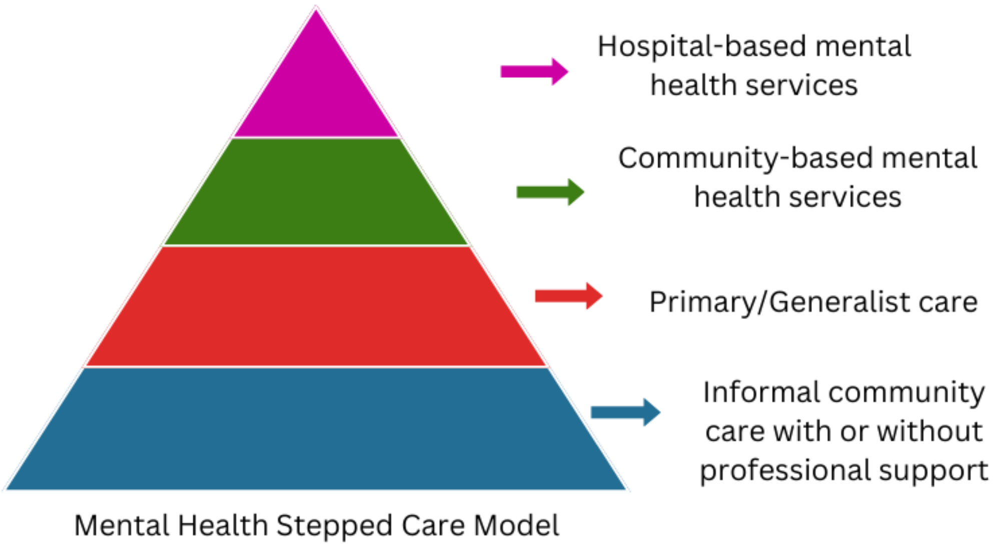

Further information on the types of care or services typically provided across the different settings, related to people from a refugee-like background is discussed below:

(1) Informal community care with or without professional support

Description

Health-promoting activities or practices undertaken by individuals, families and community members with or without support or advice from health professionals.

Service providers

Individuals, their family and friends, other community members and leaders; may be organised by community groups.

Examples

- Spiritual practice and prayer
- Group activities organised by community clubs and organisations
- Asylum Seeker Resource Centre (ASRC) community activities
- All Nations Social Cricket

(2) Primary/Generalist care

Description

Basic healthcare services are the first point of contact for routine medical needs, preventive care and health assessments.

Service providers

General practitioners (GPs); clinicians (doctors, nurses, psychologists, occupational therapists, physiotherapists) in migrant and refugee health services.

Examples

- Routine health care

- Asylum Seeker Resource Centre (ASRC)
- Health Centre NSW Refugee Health Service

(3) Community-based mental health services

Description

Specialised services addressing the mental health needs of individuals, offered outside hospitals e.g., in health facilities and clinics, patients' homes, residential care, nursing homes and assisted living accommodations.

Service providers

Community-based mental health clinician or team and community health workers.

Examples

- Community mental health centres and clinics and community-based mental health programs targeting refugees.
- NSW Service for the Treatment and Rehabilitation of Torture and Trauma Survivors (STARTTS)
- NSW Transcultural Mental Health Service

(4) Hospital-based mental health services

Description

Highly specialised treatment and mental health services provided in hospitals or specialised clinics.

Service providers

Hospital-based mental health professionals (psychiatrists, psychologists, allied health, case managers).

Examples

- Inpatient mental health units
- Child and Adolescent Health Service Refugee Health Service

Survey instructions

We would appreciate your opinion regarding the relevance and suitability of promoting the listed self-care activities for managing stress and improving mental health and wellbeing among Arabic-speaking individuals from refugee-like backgrounds in each of the four settings:

(1) informal community care with or without professional support
(2) primary/generalist care
(3) community-based mental health services
(4) hospital-based mental health services

If you do not know or feel unsure of your response, please select the 'Do not know' option.

You will also have an opportunity to provide additional comments for self-care activities that should be in the survey but are not currently included.

Delphi_R1_Community Members

Survey items

The survey items begin here.

If you are on a computer, please scroll down. If you are on a mobile device or tablet, please scroll to the side.

Indicate the relevance of practicing the following self-care activities at each of the four settings.

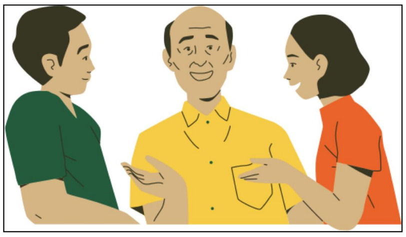

1. Having meaningful conversations with family and friends

	Very relevant	Relevant	Not relevant	Do not know
Informal community care with or without professional support	○	○	○	○
Primary/Generalist care	○	○	○	○
Community-based mental health services	○	○	○	○
Hospital-based mental health services	○	○	○	○

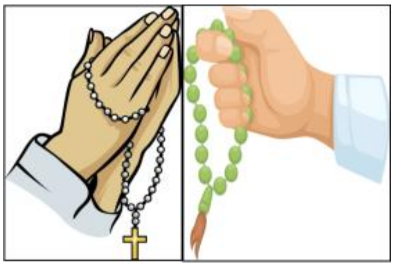

2. Praying

	Very relevant	Relevant	Not relevant	Do not know
Informal community care with or without professional support	○	○	○	○
Primary/Generalist care	○	○	○	○
Community-based mental health services	○	○	○	○
Hospital-based mental health services	○	○	○	○

Delphi_R1_Community Members

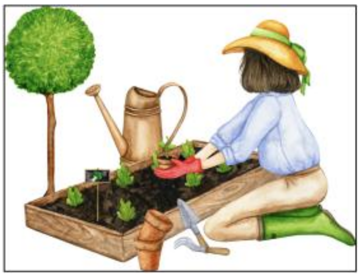

3. Gardening

	Very relevant	Relevant	Not relevant	Do not know
Informal community care with or without professional support				
Primary/Generalist care				
Community-based mental health services				
Hospital-based mental health services				

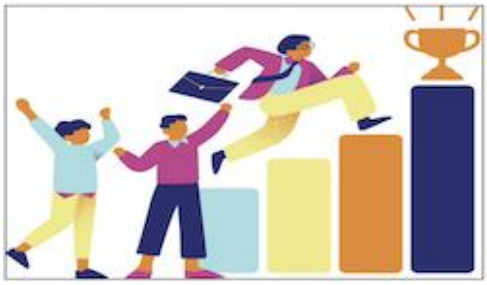

4. Having personal or professional goals and working towards them

	Very relevant	Relevant	Not relevant	Do not know
Informal community care with or without professional support				
Primary/Generalist care				
Community-based mental health services				
Hospital-based mental health services				

Delphi_R1_Community Members

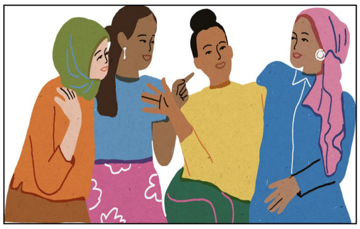

5. Spending time with people of the same culture or religion

	Very relevant	Relevant	Not relevant	Do not know
Informal community care with or without professional support	○	○	○	○
Primary/Generalist care	○	○	○	○
Community-based mental health services	○	○	○	○
Hospital-based mental health services	○	○	○	○

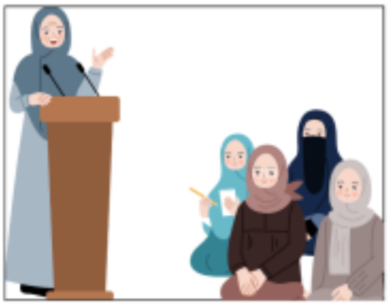

6. Attending religious services

	Very relevant	Relevant	Not relevant	Do not know
Informal community care with or without professional support	○	○	○	○
Primary/Generalist care	○	○	○	○
Community-based mental health services	○	○	○	○
Hospital-based mental health services	○	○	○	○

Delphi_R1_Community Members

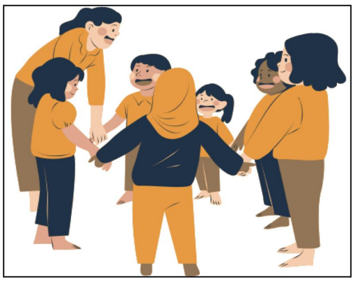

7. Playing with children

	Very relevant	Relevant	Not relevant	Do not know
Informal community care with or without professional support	○	○	○	○
Primary/Generalist care	○	○	○	○
Community-based mental health services	○	○	○	○
Hospital-based mental health services	○	○	○	○

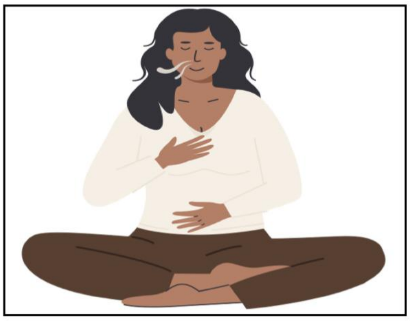

8. Practicing deep breathing

	Very relevant	Relevant	Not relevant	Do not know
Informal community care with or without professional support	○	○	○	○
Primary/Generalist care	○	○	○	○
Community-based mental health services	○	○	○	○
Hospital-based mental health services	○	○	○	○

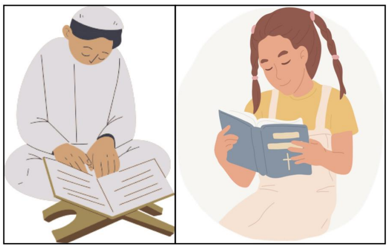

9. Reading or listening to verses from the Holy books or other religious writings

	Very relevant	Relevant	Not relevant	Do not know
Informal community care with or without professional support	○	○	○	○
Primary/Generalist care	○	○	○	○
Community-based mental health services	○	○	○	○
Hospital-based mental health services	○	○	○	○

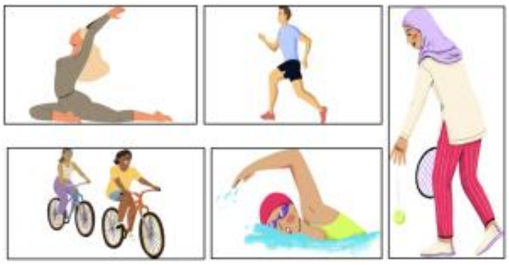

10. Physical activity (e.g., walking, hiking, jogging, aerobics and fitness exercises, yoga, playing sports, cycling, swimming, going to the gym)

	Very relevant	Relevant	Not relevant	Do not know
Informal community care with or without professional support	○	○	○	○
Primary/Generalist care	○	○	○	○
Community-based mental health services	○	○	○	○
Hospital-based mental health services	○	○	○	○

Delphi_R1_Community Members

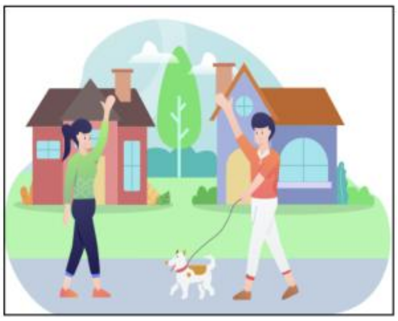

11. Connecting with neighbours and community members

	Very relevant	Relevant	Not relevant	Do not know
Informal community care with or without professional support	◯	◯	◯	◯
Primary/Generalist care	◯	◯	◯	◯
Community-based mental health services	◯	◯	◯	◯
Hospital-based mental health services	◯	◯	◯	◯

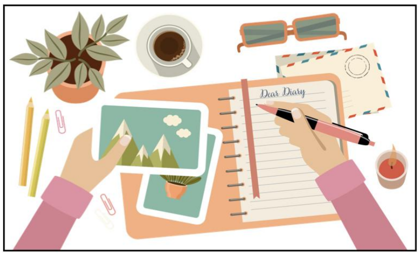

12. Keeping a diary to reflect on daily situations in life

	Very relevant	Relevant	Not relevant	Do not know
Informal community care with or without professional support	◯	◯	◯	◯
Primary/Generalist care	◯	◯	◯	◯
Community-based mental health services	◯	◯	◯	◯
Hospital-based mental health services	◯	◯	◯	◯

Delphi_R1_Community Members

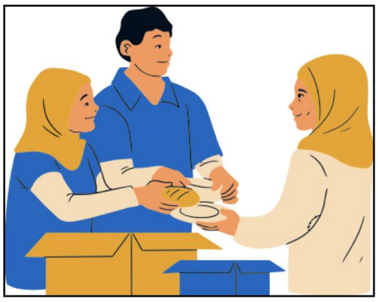

13. Volunteering and doing community service

	Very relevant	Relevant	Not relevant	Do not know
Informal community care with or without professional support	◯	◯	◯	◯
Primary/Generalist care	◯	◯	◯	◯
Community-based mental health services	◯	◯	◯	◯
Hospital-based mental health services	◯	◯	◯	◯

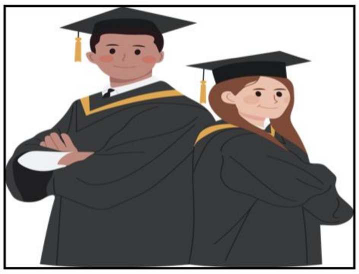

14. Studying and pursuing an education

	Very relevant	Relevant	Not relevant	Do not know
Informal community care with or without professional support	◯	◯	◯	◯
Primary/Generalist care	◯	◯	◯	◯
Community-based mental health services	◯	◯	◯	◯
Hospital-based mental health services	◯	◯	◯	◯

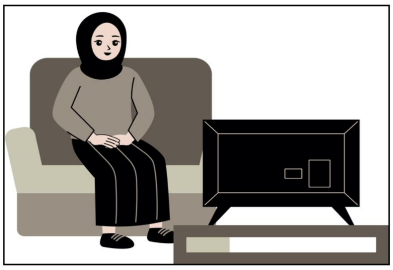

15. Watching television

	Very relevant	Relevant	Not relevant	Do not know
Informal community care with or without professional support	○	○	○	○
Primary/Generalist care	○	○	○	○
Community-based mental health services	○	○	○	○
Hospital-based mental health services	○	○	○	○

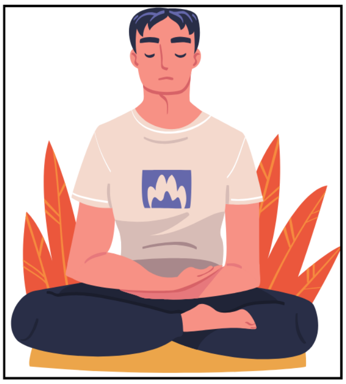

16. Practicing mindfulness

	Very relevant	Relevant	Not relevant	Do not know
Informal community care with or without professional support	○	○	○	○
Primary/Generalist care	○	○	○	○
Community-based mental health services	○	○	○	○
Hospital-based mental health services	○	○	○	○

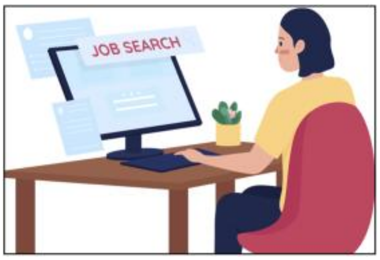

17. Finding work

	Very relevant	Relevant	Not relevant	Do not know
Informal community care with or without professional support	○	○	○	○
Primary/Generalist care	○	○	○	○
Community-based mental health services	○	○	○	○
Hospital-based mental health services	○	○	○	○

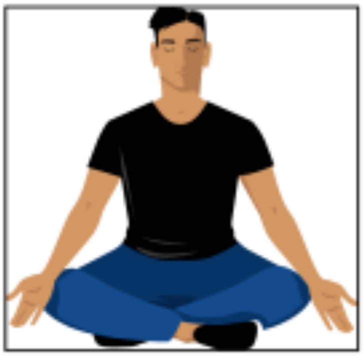

18. Practicing meditation

	Very relevant	Relevant	Not relevant	Do not know
Informal community care with or without professional support	○	○	○	○
Primary/Generalist care	○	○	○	○
Community-based mental health services	○	○	○	○
Hospital-based mental health services	○	○	○	○

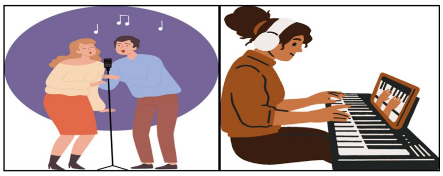

19. Pursuing performing arts (e.g., acting, theatre and drama, storytelling, singing, playing musical instruments, dancing)

	Very relevant	Relevant	Not relevant	Do not know
Informal community care with or without professional support	○	○	○	○
Primary/Generalist care	○	○	○	○
Community-based mental health services	○	○	○	○
Hospital-based mental health services	○	○	○	○

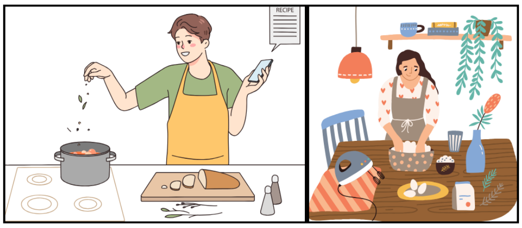

20. Cooking and baking

	Very relevant	Relevant	Not relevant	Do not know
Informal community care with or without professional support	○	○	○	○
Primary/Generalist care	○	○	○	○
Community-based mental health services	○	○	○	○
Hospital-based mental health services	○	○	○	○

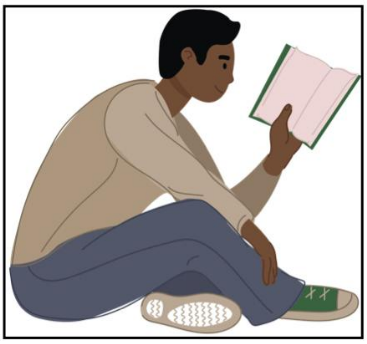

21. Reading

	Very relevant	Relevant	Not relevant	Do not know
Informal community care with or without professional support	◯	◯	◯	◯
Primary/Generalist care	◯	◯	◯	◯
Community-based mental health services	◯	◯	◯	◯
Hospital-based mental health services	◯	◯	◯	◯

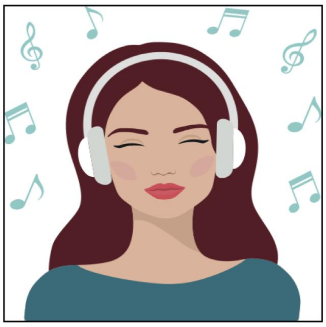

22. Listening to music

	Very relevant	Relevant	Not relevant	Do not know
Informal community care with or without professional support	◯	◯	◯	◯
Primary/Generalist care	◯	◯	◯	◯
Community-based mental health services	◯	◯	◯	◯
Hospital-based mental health services	◯	◯	◯	◯

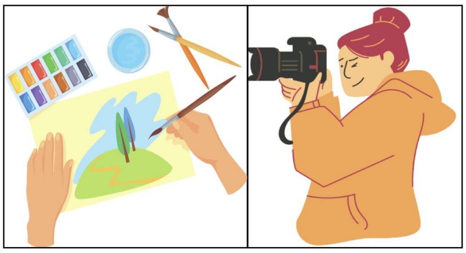

23. Pursuing visual arts (e.g., drawing and painting, photography, filmmaking, graphic design)

	Very relevant	Relevant	Not relevant	Do not know
Informal community care with or without professional support				
Primary/Generalist care				
Community-based mental health services				
Hospital-based mental health services				

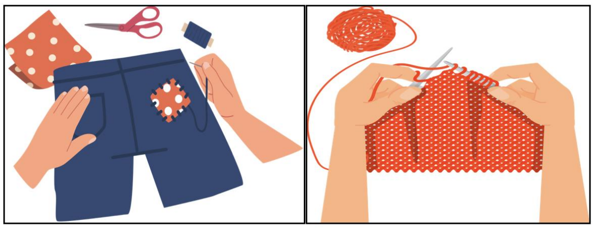

24. Doing crafts (e.g., making cards, sewing, knitting)

	Very relevant	Relevant	Not relevant	Do not know
Informal community care with or without professional support				
Primary/Generalist care				
Community-based mental health services				
Hospital-based mental health services				

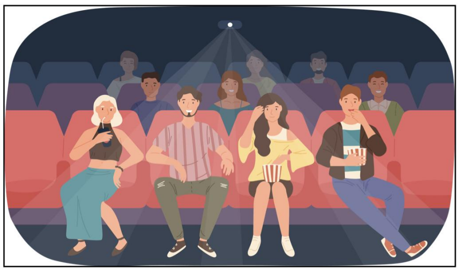

25. Going to the cinema

	Very relevant	Relevant	Not relevant	Do not know
Informal community care with or without professional support	○	○	○	○
Primary/Generalist care	○	○	○	○
Community-based mental health services	○	○	○	○
Hospital-based mental health services	○	○	○	○

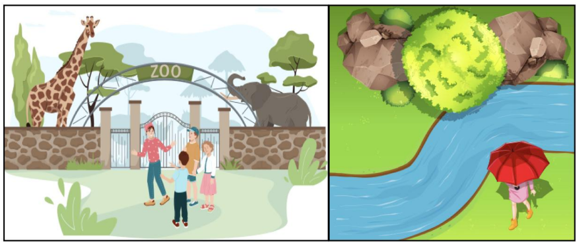

26. Connecting with nature (e.g., going to parks, the zoo, beaches, outdoor picnics, riverbanks, birdwatching)

	Very relevant	Relevant	Not relevant	Do not know
Informal community care with or without professional support	○	○	○	○
Primary/Generalist care	○	○	○	○
Community-based mental health services	○	○	○	○
Hospital-based mental health services	○	○	○	○

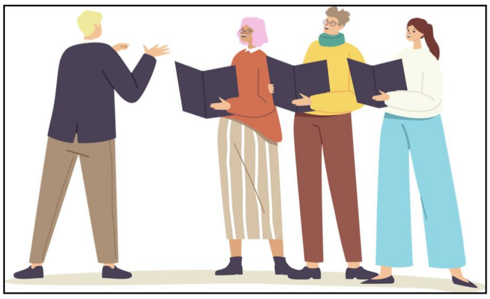

27. Singing in a choir

	Very relevant	Relevant	Not relevant	Do not know
Informal community care with or without professional support	◯	◯	◯	◯
Primary/Generalist care	◯	◯	◯	◯
Community-based mental health services	◯	◯	◯	◯
Hospital-based mental health services	◯	◯	◯	◯

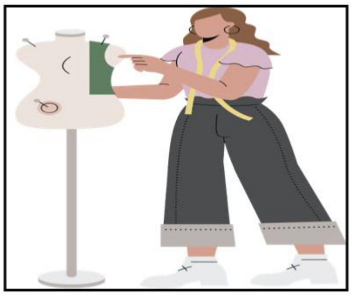

28. Designing clothes

	Very relevant	Relevant	Not relevant	Do not know
Informal community care with or without professional support	◯	◯	◯	◯
Primary/Generalist care	◯	◯	◯	◯
Community-based mental health services	◯	◯	◯	◯
Hospital-based mental health services	◯	◯	◯	◯

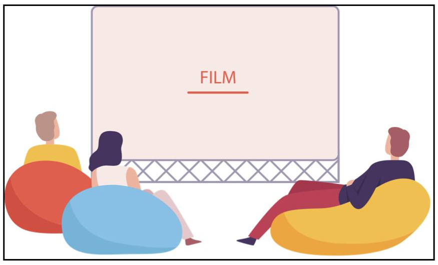

29. Watching movies

	Very relevant	Relevant	Not relevant	Do not know
Informal community care with or without professional support	○	○	○	○
Primary/Generalist care	○	○	○	○
Community-based mental health services	○	○	○	○
Hospital-based mental health services	○	○	○	○

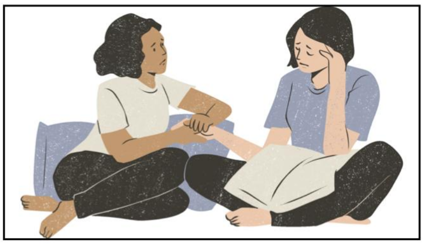

30. Discussing problems with friends, family and trusted others

	Very relevant	Relevant	Not relevant	Do not know
Informal community care with or without professional support	○	○	○	○
Primary/Generalist care	○	○	○	○
Community-based mental health services	○	○	○	○
Hospital-based mental health services	○	○	○	○

Delphi_R1_Community Members

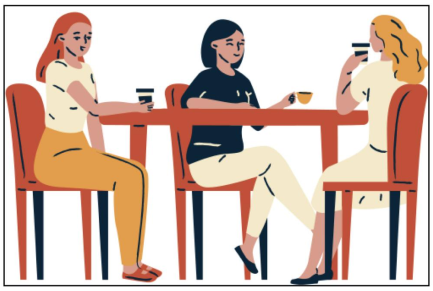

31. Making friends

	Very relevant	Relevant	Not relevant	Do not know
Informal community care with or without professional support	◯	◯	◯	◯
Primary/Generalist care	◯	◯	◯	◯
Community-based mental health services	◯	◯	◯	◯
Hospital-based mental health services	◯	◯	◯	◯

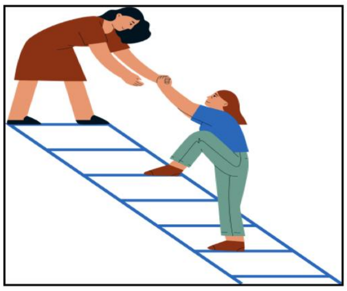

32. Asking for help from family, friends and trusted others

	Very relevant	Relevant	Not relevant	Do not know
Informal community care with or without professional support	◯	◯	◯	◯
Primary/Generalist care	◯	◯	◯	◯
Community-based mental health services	◯	◯	◯	◯
Hospital-based mental health services	◯	◯	◯	◯

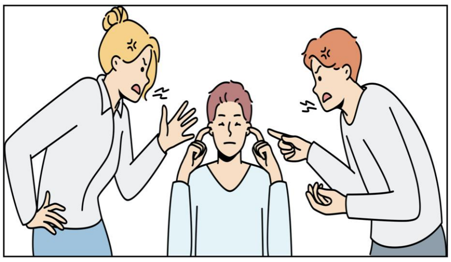

33. Taking a break from stressful situations and negative people

	Very relevant	Relevant	Not relevant	Do not know
Informal community care with or without professional support				
Primary/Generalist care				
Community-based mental health services				
Hospital-based mental health services				

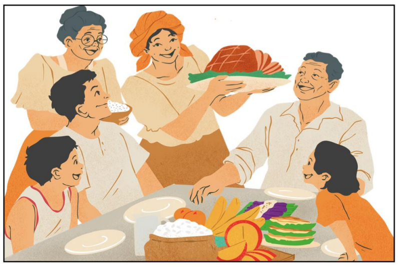

34. Attending cultural or ethnic festivals and events

	Very relevant	Relevant	Not relevant	Do not know
Informal community care with or without professional support				
Primary/Generalist care				
Community-based mental health services				
Hospital-based mental health services				

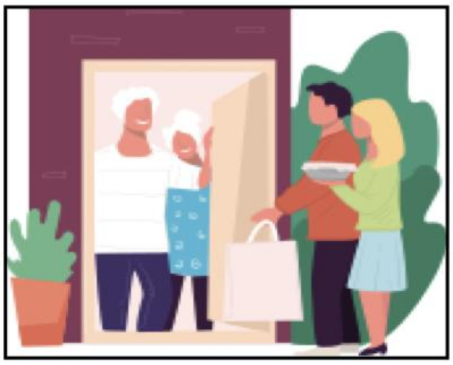

35. Visiting family and friends

	Very relevant	Relevant	Not relevant	Do not know
Informal community care with or without professional support	○	○	○	○
Primary/Generalist care	○	○	○	○
Community-based mental health services	○	○	○	○
Hospital-based mental health services	○	○	○	○

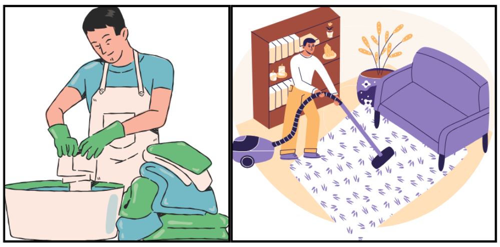

36. Doing housework

	Very relevant	Relevant	Not relevant	Do not know
Informal community care with or without professional support	○	○	○	○
Primary/Generalist care	○	○	○	○
Community-based mental health services	○	○	○	○
Hospital-based mental health services	○	○	○	○

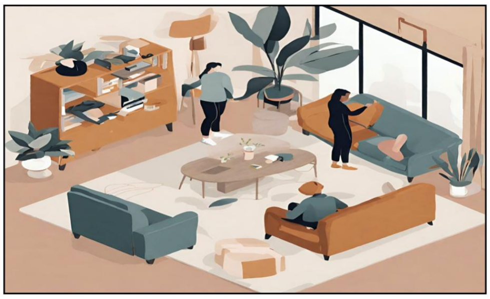

37. Changing house décor

	Very relevant	Relevant	Not relevant	Do not know
Informal community care with or without professional support	○	○	○	○
Primary/Generalist care	○	○	○	○
Community-based mental health services	○	○	○	○
Hospital-based mental health services	○	○	○	○

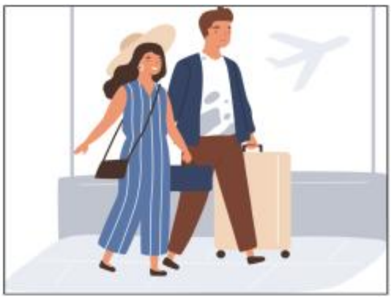

38. Travelling

	Very relevant	Relevant	Not relevant	Do not know
Informal community care with or without professional support	○	○	○	○
Primary/Generalist care	○	○	○	○
Community-based mental health services	○	○	○	○
Hospital-based mental health services	○	○	○	○

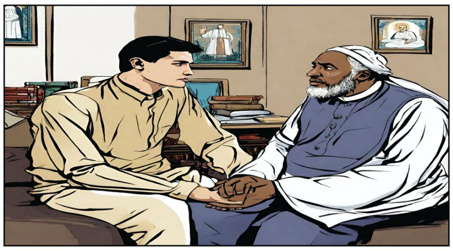

39. Seeking help from religious leaders

	Very relevant	Relevant	Not relevant	Do not know
Informal community care with or without professional support	○	○	○	○
Primary/Generalist care	○	○	○	○
Community-based mental health services	○	○	○	○
Hospital-based mental health services	○	○	○	○

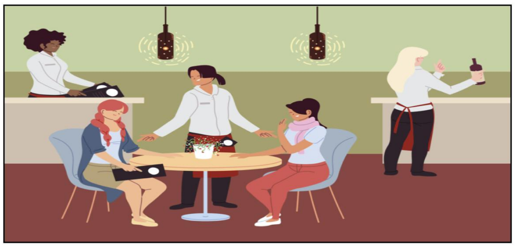

40. Going to cafes and restaurants with family or friends

	Very relevant	Relevant	Not relevant	Do not know
Informal community care with or without professional support	○	○	○	○
Primary/Generalist care	○	○	○	○
Community-based mental health services	○	○	○	○
Hospital-based mental health services	○	○	○	○

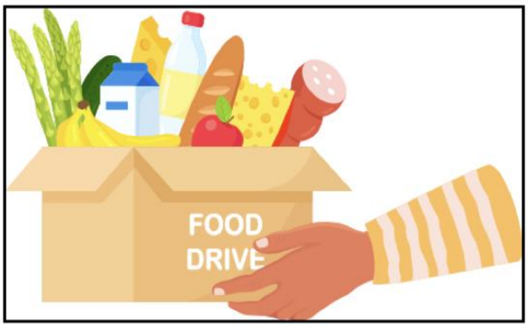

41. Performing acts of service and charity

	Very relevant	Relevant	Not relevant	Do not know
Informal community care with or without professional support	○	○	○	○
Primary/Generalist care	○	○	○	○
Community-based mental health services	○	○	○	○
Hospital-based mental health services	○	○	○	○

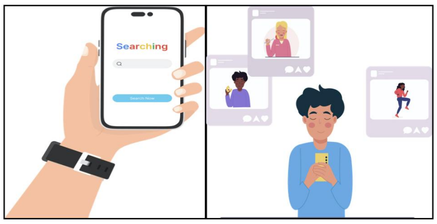

42. Browsing the internet and social media

	Very relevant	Relevant	Not relevant	Do not know
Informal community care with or without professional support	○	○	○	○
Primary/Generalist care	○	○	○	○
Community-based mental health services	○	○	○	○
Hospital-based mental health services	○	○	○	○

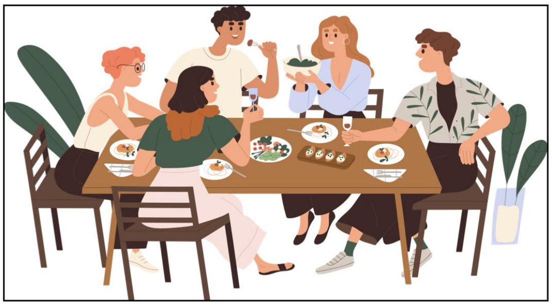

43. Going to social gatherings and parties

	Very relevant	Relevant	Not relevant	Do not know
Informal community care with or without professional support	◯	◯	◯	◯
Primary/Generalist care	◯	◯	◯	◯
Community-based mental health services	◯	◯	◯	◯
Hospital-based mental health services	◯	◯	◯	◯

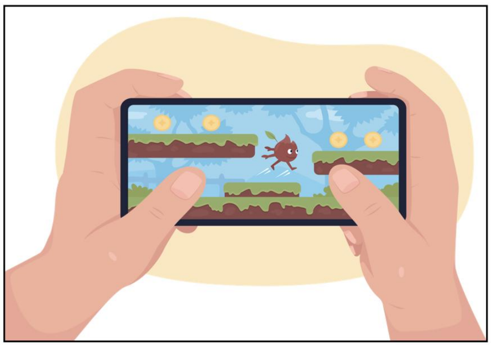

44. Playing electronic games (e.g., mobile games)

	Very relevant	Relevant	Not relevant	Do not know
Informal community care with or without professional support	◯	◯	◯	◯
Primary/Generalist care	◯	◯	◯	◯
Community-based mental health services	◯	◯	◯	◯
Hospital-based mental health services	◯	◯	◯	◯

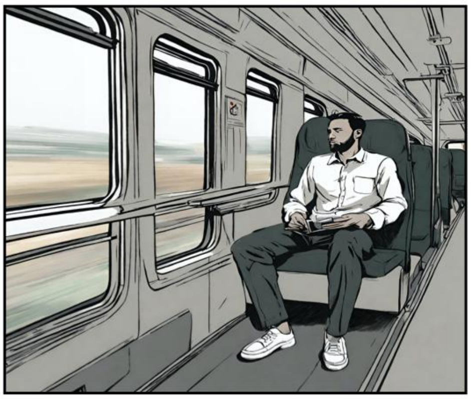

45. Taking train trips

	Very relevant	Relevant	Not relevant	Do not know
Informal community care with or without professional support	○	○	○	○
Primary/Generalist care	○	○	○	○
Community-based mental health services	○	○	○	○
Hospital-based mental health services	○	○	○	○

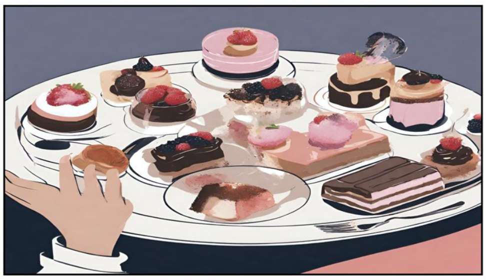

46. Eating sweets

	Very relevant	Relevant	Not relevant	Do not know
Informal community care with or without professional support	○	○	○	○
Primary/Generalist care	○	○	○	○
Community-based mental health services	○	○	○	○
Hospital-based mental health services	○	○	○	○

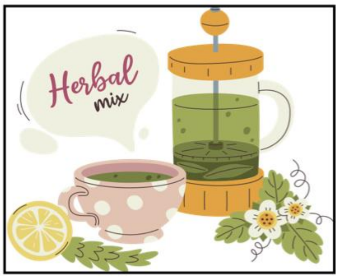

47. Drinking herbal tea

	Very relevant	Relevant	Not relevant	Do not know
Informal community care with or without professional support	○	○	○	○
Primary/Generalist care	○	○	○	○
Community-based mental health services	○	○	○	○
Hospital-based mental health services	○	○	○	○

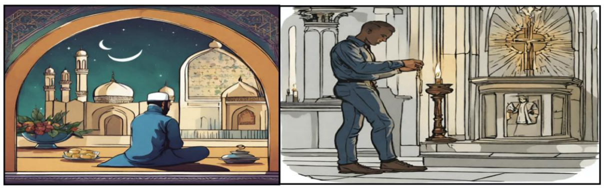

48. Observing religious rituals

	Very relevant	Relevant	Not relevant	Do not know
Informal community care with or without professional support	○	○	○	○
Primary/Generalist care	○	○	○	○
Community-based mental health services	○	○	○	○
Hospital-based mental health services	○	○	○	○

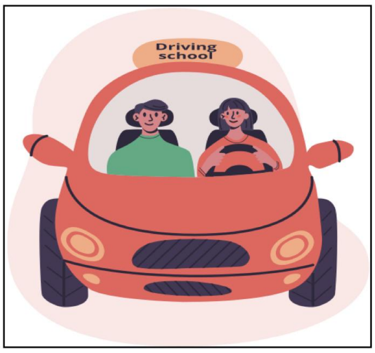

49. Learning to drive

	Very relevant	Relevant	Not relevant	Do not know
Informal community care with or without professional support	○	○	○	○
Primary/Generalist care	○	○	○	○
Community-based mental health services	○	○	○	○
Hospital-based mental health services	○	○	○	○

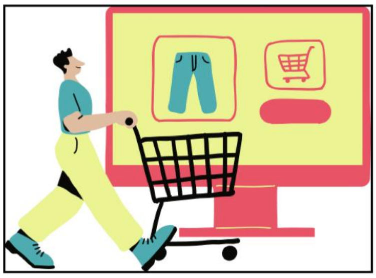

50. Shopping

	Very relevant	Relevant	Not relevant	Do not know
Informal community care with or without professional support	○	○	○	○
Primary/Generalist care	○	○	○	○
Community-based mental health services	○	○	○	○
Hospital-based mental health services	○	○	○	○

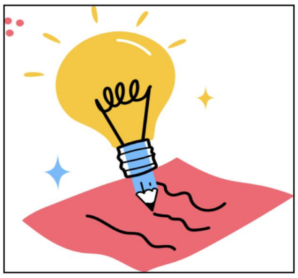

51. Problem-solving by writing down the problem and thinking about the best solution

	Very relevant	Relevant	Not relevant	Do not know
Informal community care with or without professional support				
Primary/Generalist care				
Community-based mental health services				
Hospital-based mental health services				

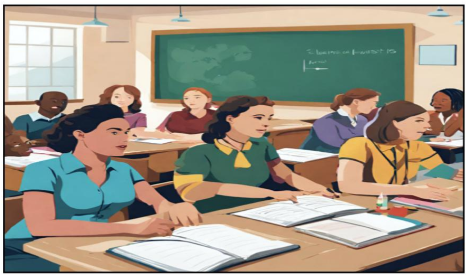

52. Learning English

	Very relevant	Relevant	Not relevant	Do not know
Informal community care with or without professional support				
Primary/Generalist care				
Community-based mental health services				
Hospital-based mental health services				

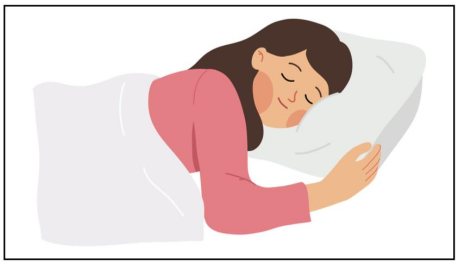

53. Sleeping

	Very relevant	Relevant	Not relevant	Do not know
Informal community care with or without professional support	○	○	○	○
Primary/Generalist care	○	○	○	○
Community-based mental health services	○	○	○	○
Hospital-based mental health services	○	○	○	○

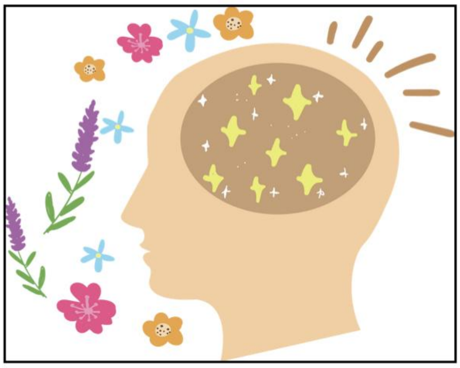

54. Reframing thoughts into realistic and positive ideas and beliefs

	Very relevant	Relevant	Not relevant	Do not know
Informal community care with or without professional support	○	○	○	○
Primary/Generalist care	○	○	○	○
Community-based mental health services	○	○	○	○
Hospital-based mental health services	○	○	○	○

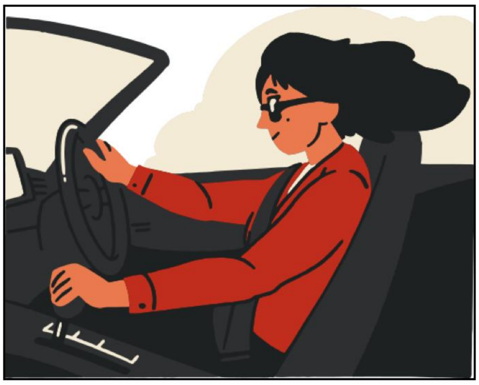

55. Going for drives

	Very relevant	Relevant	Not relevant	Do not know
Informal community care with or without professional support	○	○	○	○
Primary/Generalist care	○	○	○	○
Community-based mental health services	○	○	○	○
Hospital-based mental health services	○	○	○	○

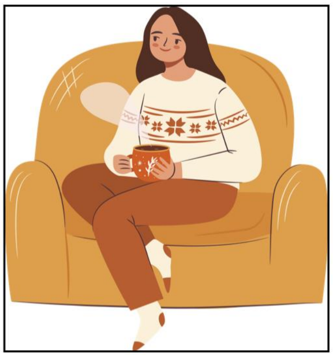

56. Spending ‘me’ time

	Very relevant	Relevant	Not relevant	Do not know
Informal community care with or without professional support	○	○	○	○
Primary/Generalist care	○	○	○	○
Community-based mental health services	○	○	○	○
Hospital-based mental health services	○	○	○	○

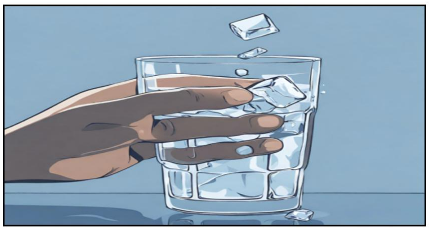

57. Having a cold drink to calm oneself

	Very relevant	Relevant	Not relevant	Do not know
Informal community care with or without professional support	○	○	○	○
Primary/Generalist care	○	○	○	○
Community-based mental health services	○	○	○	○
Hospital-based mental health services	○	○	○	○

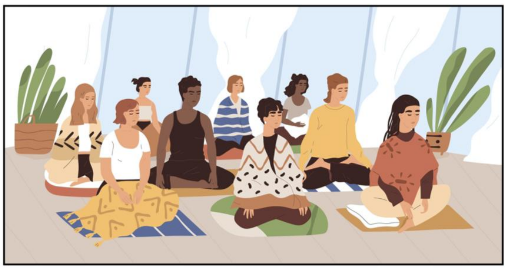

58. Going to stress management workshops and short courses

	Very relevant	Relevant	Not relevant	Do not know
Informal community care with or without professional support	○	○	○	○
Primary/Generalist care	○	○	○	○
Community-based mental health services	○	○	○	○
Hospital-based mental health services	○	○	○	○

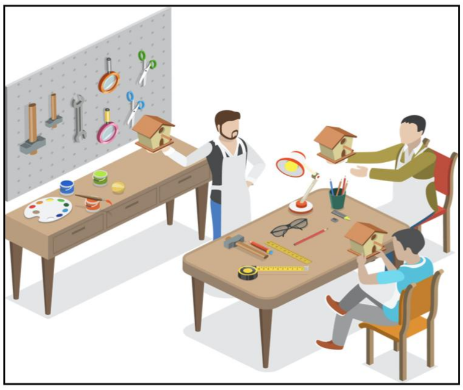

59. Pursuing vocational training (e.g., TAFE courses)

	Very relevant	Relevant	Not relevant	Do not know
Informal community care with or without professional support	○	○	○	○
Primary/Generalist care	○	○	○	○
Community-based mental health services	○	○	○	○
Hospital-based mental health services	○	○	○	○

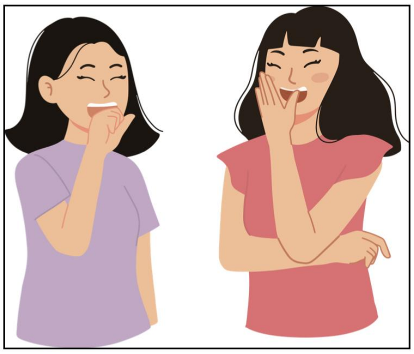

60. Joking and laughing with friends

	Very relevant	Relevant	Not relevant	Do not know
Informal community care with or without professional support	○	○	○	○
Primary/Generalist care	○	○	○	○
Community-based mental health services	○	○	○	○
Hospital-based mental health services	○	○	○	○

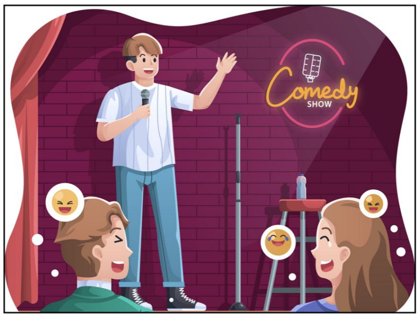

61. Watching comedy shows, funny videos or children’s cartoons

	Very relevant	Relevant	Not relevant	Do not know
Informal community care with or without professional support	○	○	○	○
Primary/Generalist care	○	○	○	○
Community-based mental health services	○	○	○	○
Hospital-based mental health services	○	○	○	○

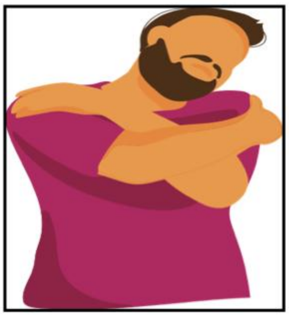

62. Learning to accept situations in life that cannot be changed

	Very relevant	Relevant	Not relevant	Do not know
Informal community care with or without professional support	○	○	○	○
Primary/Generalist care	○	○	○	○
Community-based mental health services	○	○	○	○
Hospital-based mental health services	○	○	○	○

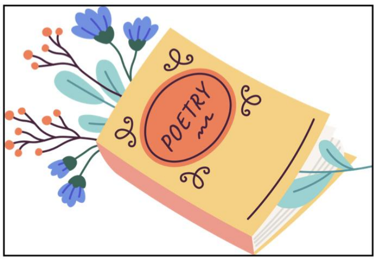

63. Reading or writing poetry

	Very relevant	Relevant	Not relevant	Do not know
Informal community care with or without professional support	○	○	○	○
Primary/Generalist care	○	○	○	○
Community-based mental health services	○	○	○	○
Hospital-based mental health services	○	○	○	○

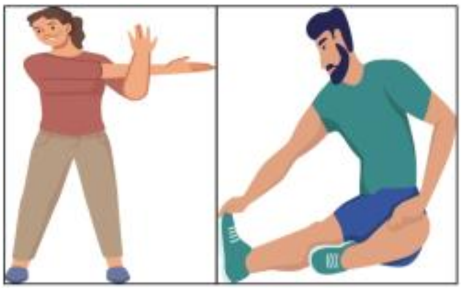

64. Practicing muscle relaxation exercises

	Very relevant	Relevant	Not relevant	Do not know
Informal community care with or without professional support	○	○	○	○
Primary/Generalist care	○	○	○	○
Community-based mental health services	○	○	○	○
Hospital-based mental health services	○	○	○	○

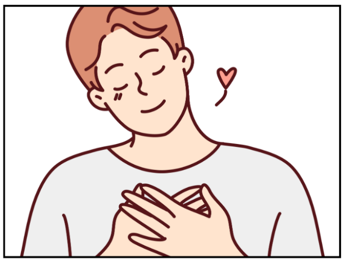

65. Being thankful for positive life events

	Very relevant	Relevant	Not relevant	Do not know
Informal community care with or without professional support	○	○	○	○
Primary/Generalist care	○	○	○	○
Community-based mental health services	○	○	○	○
Hospital-based mental health services	○	○	○	○

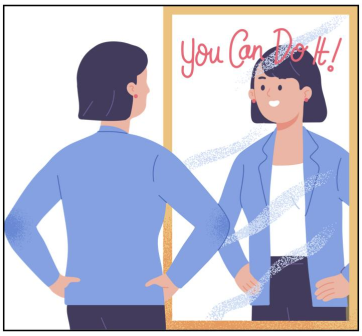

66. Engaging in positive self-talk (e.g., writing positive affirmation statements)

	Very relevant	Relevant	Not relevant	Do not know
Informal community care with or without professional support	○	○	○	○
Primary/Generalist care	○	○	○	○
Community-based mental health services	○	○	○	○
Hospital-based mental health services	○	○	○	○

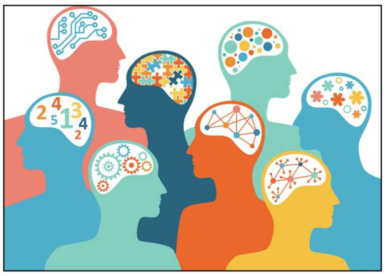

67. Learning to consider different perspectives and accepting other people's views

	Very relevant	Relevant	Not relevant	Do not know
Informal community care with or without professional support	○	○	○	○
Primary/Generalist care	○	○	○	○
Community-based mental health services	○	○	○	○
Hospital-based mental health services	○	○	○	○

Please provide your comments on any self-care activity that should be in the survey but not currently included.

End of survey

Thank you for your effort and time in completing the survey.

Your valuable input is greatly appreciated and will contribute significantly to our research.

We will be in touch soon for the subsequent rounds 2 and 3 of the survey.

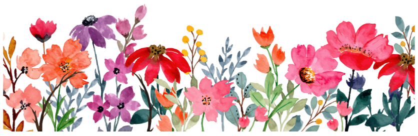

Delphi_R1_HealthProfessionals

Project Title: Recommendations for mental health self-care strategies among Arabic-speaking community members from refugee-like backgrounds living in Australia: A Delphi study.

First Round Delphi Survey

Participant Information Sheet

Project Summary:
This study aims to develop a set of mental health self-care recommendations for Arabic-speaking community from refugee-like backgrounds living in Australia. The purpose is to gather opinions on mental health self-care strategies from two groups of people: health professionals who have experience working with the target group (practitioners and researchers and academics), and Arabic-speaking community members and leaders. You have been invited to participate in the health professional panel.

Participants are required to meet the eligibility criteria:

- Qualified health professionals
- Have at least 2 years full-time or equivalent part-time experience
- Have experience working with Arabic-speaking individuals from refugee-like backgrounds

What will I be asked to do?
You will be required to complete three rounds of an online survey. Each round will be opened for two weeks.

In each survey round, you will be asked to give your opinion regarding the relevance and suitability of the listed self-care activities for managing stress and improving mental health among Arabic-speaking populations from refugee-like backgrounds, across the four settings in the Australian Mental Health Stepped Care Model. The response options are: ‘Very relevant’, ‘Relevant’, ‘Not relevant' and ‘Do not know’. You may choose multiple settings for a single self-care activity.

Open-ended comment boxes will be available to allow you to provide specific feedback regarding any new self-care activity that is not included in the survey.

How much of my time will I need to give?
Each survey round will take approximately 60 minutes to complete.

Can I withdraw from the study?
Your participation is entirely voluntary and you are not obliged to be involved. If you do participate, you can withdraw at any time without giving reason and without any impact on your relationships with the researchers or partner organisations. However, once the survey round has closed, individual data will no longer be able to be withdrawn from the survey. All survey data will be de-identified.

You can download and read the Participant Information Sheet here.

Do you meet the eligibility criteria?

- Yes
- No

Delphi_R1_HealthProfessionals

Consent

I hereby consent to participate in the above named research project.

I acknowledge that:

- I have read the participant information sheet and have been given the opportunity to discuss the information and my involvement in the project with the researcher/s.
- The procedures required for the project and the time involved have been explained to me, and any questions I have about the project have been answered to my satisfaction.
- I understand that my involvement is confidential and that the information gained during the study may be published and stored for other research use but no information about me will be used in any way that reveals my identity.
- I understand that my participation in this study will have no effect on my relationship with the researcher/s, and any organisations involved, now or in the future. I understand that participation is entirely voluntary and I am not obliged to be involved.
- If I do participate, I can withdraw at any time without giving reason. I understand that I am able to withdraw my data and information within the time (two weeks) that each survey is opened and that once the survey closes I will be unable to withdraw my data and information given within that survey round from this project.

I consent to participate in this project

- Yes
- No

I consent to providing my data to be used in other related projects for an extended period of time

- Yes
- No

Demographic Information

We are collecting your name and email address for sending the subsequent rounds of survey and a summary of results from the previous round. This information will only be accessible to the research team. Once this study is completed, the file containing participants' names and email addresses will be deleted permanently.

First name Phone number Email address

What is your age?

- 18–24
- 25–34
- 35–44
- 45–54
- 55–64
- 65 or older
- Prefer not to say

Delphi_R1_HealthProfessionals

What is your gender?
- Woman
- Man
- Prefer not to say

Which of the following best describes your religious beliefs?
- Muslim
- Christian
- Mandaean
- Other (please specify)
- Prefer not to say

What is your country of birth?
- Country of birth
- Prefer not to say

Which languages do you speak? (You can choose multiple options)
- English
- Arabic
- Other (please specify)
- Prefer not to say

How many years of professional experience do you have?
- More than 2 years, less than 5 years
- More than 5 years

What is your discipline? (You can choose multiple options)
- Psychology
- Psychiatry
- Medicine
- Nursing
- Physiotherapy
- Occupational therapy
- Other (please specify)

What is the main sector you work in? (You can choose multiple options)
- Government
- Non-governmental organisation
- Private practice
- Other (please specify)
- Prefer not to say
